# Effect of arthroscopic rotator cuff surgery in patients with preoperative restricted range of motion

**DOI:** 10.1186/s12891-016-0956-4

**Published:** 2016-02-24

**Authors:** Helen Razmjou, Patrick Henry, Giuseppe Costa, Tim Dwyer, Richard Holtby

**Affiliations:** Department of Rehabilitation, Holland Orthopedic & Arthritic Centre, Sunnybrook Health Sciences Centre, Toronto, Canada; Department of Physical therapy, Faculty of Medicine, University of Toronto, Toronto, Canada; Division of Orthopedic Surgery, Department of Surgery, Holland Orthopedic & Arthritic Centre, Sunnybrook Health Sciences Centre, Toronto, Canada; Division of Orthopaedic Surgery, Department of Surgery, Faculty of Medicine, University of Toronto, Toronto, Canada; Division of Orthopedic Surgery, Department of Surgery, Toronto East General Hospital, Toronto, Canada; Division of Orthopedic Surgery, Department of Surgery, Women’s College and Mt Sinai Hospitals, Toronto, Canada

**Keywords:** Shoulder, Pre-operative Stiffness, Rotator Cuff

## Abstract

**Background:**

The purpose of this study was to examine the impact of rotator cuff (RC) decompression and/or repair on post-operative ROM in patients with pre-operative restricted passive motion who had undergone arthroscopic subacromial debridement and/or rotator cuff repair. Potential predictors of ROM recovery such as age, sex, mechanism of injury, type of surgery, presence of an endocrine illness and having an active Worker Compensation claim related to the shoulder were explored.

**Methods:**

A retrospective analysis of prospectively collected data was performed. Pre-operative stiffness measured intra-operatively was defined as flexion of < =100° or external rotation of < =30° under anesthesia. Patients who received manipulation under anesthesia or required capsular release were excluded.

**Results:**

Two hundred and eighteen patients met the criteria for having stiffness under anesthesia. Twenty six patients had stiffness in both directions, 19 patients had isolated restricted flexion and 173 had isolated restricted external rotation. At six months post-operatively, a statistically significant improvement was observed on average in all disability measures (*P <* 0.0001). The ROM improved on average in the restricted direction at 6 months (*p <* 0.0001). Older age had a negative impact on recovery of external rotation (F_2,216_ = −5.78, *p =* 0.02). Being a female, having a traumatic event, having a RC repair, or suffering from an endocrine illness such as diabetes, did not have a negative impact on recovery. Patients with an active work-related compensation claim showed an inferior recovery of flexion (F_2,216_ = −8.76, *p =* 0.003).

**Conclusion:**

Patients with RC pathology and concomitant stiffness showed significant improvement in ROM at six months following RC decompression or repair without the need for formal capsular releases or the performance of manipulation under anesthesia. Older patients and those with active Workers Compensation claim showed an inferior recovery in isolated directions.

## Background

Rotator cuff pathology is a common and disabling condition [1] that affects function and overall quality of life [2–6]. Surgical interventions to address rotator cuff pathology have been the subject of investigations for years, and both rotator cuff decompression [7–9] and repair have shown successful results [10–12]. However, the degree to which pre-operative stiffness improves following rotator cuff surgery has not been extensively examined. The limited research in this area has shown that manipulation under anesthesia or capsular release in association with surgical treatment of the rotator cuff pathology can facilitate recovery of range of motion (ROM) [13–18]. The only study that has examined the impact of rotator cuff surgery without manipulation under anesthesia or capsular release is by Tauro [19], who reported that patients with mild and moderate stiffness responded well to rotator cuff repair alone, but suggested that those with major total loss of motion (>70 °) required interventions addressing the capsular tightness.

The purpose of this study was to examine the impact of rotator cuff decompression and/or repair on post-operative ROM and disability in patients with pre-operative restricted passive motion. It was hypothesized that 1) stiffness would improve following appropriate surgical treatment of rotator cuff pathology, and 2) patient demographics (age, sex, mechanism of injury, an endocrine condition (diabetes, thyroid or hypothyroid) and workers’ compensation claim would have an impact on recovery.

## Methods

### Subjects

Prospectively collected data of patients with rotator cuff pathology who had participated in previous formal studies from 2004 to 2014 were used for data analysis. As part of a standard protocol, all patients had completed a pre-operative course of conservative treatment for a minimum of six months, which included physiotherapy, anti-inflammatory medication, or cortisone injection before being considered for surgery. All patients had provided written informed consent for participation in the original studies. Approval for using the existent data was obtained from the Research Ethics Board of the Sunnybrook Health Sciences Centre (Project ID# 287–2014).

Inclusion criteria included a diagnosis of impingement syndrome and/or a partial thickness rotator cuff tear requiring decompression surgery (acromioplasty or resection of lateral clavicle), or full-thickness rotator cuff tear requiring arthroscopic repair. Patients who had shoulder stiffness due to concomitant osteoarthritis of the glenohumeral joint were excluded. Patients with typical presentation of adhesive capsulitis (absence of significant rotator cuff pathology in combination with capsular pattern of stiffness, which involves external rotation of 0 to 10° in association with significant flexion loss), who received manipulation under anesthesia or required capsular release were also excluded.

Patients with stiffness of the shoulder in either flexion or external rotation as measured by a ROM exam under anesthesia were identified. Stiffness was defined as passive flexion of < =100° or passive external rotation (in neutral position) of < =30°. Patients were divided into two groups based on the direction of shoulder stiffness. Group 1 had restriction of motion in both directions (flexion and external rotation), while Group 2 had restriction of motion only in one direction. Group 2 was divided into two subcategories: 2a) restricted flexion only and, 2b) restricted external rotation only.

### Outcome measures

Outcomes were assessed at the six month post-operative office visit. The primary outcome measure was post-operative passive ROM in flexion and external rotation in neutral (0° of abduction). The American Shoulder and Elbow Surgeons (ASES) form [20], the Constant Murley Score (CMS) [21], and the Short Western Ontario Rotator Cuff (ShortWORC) index [22] were utilized as secondary outcome measures. All subjective measures of disability have been reported to be reliable and valid in patients with rotator cuff pathology [21, 23–26].

### Rehabilitation

All patients followed a standardized rehabilitation protocol based on type of surgery. Patients who had decompression started active assisted ROM on post-day one and progressed to isometric, active and strengthening within 1–3 weeks. Patients who underwent RC repair remained in an ultrasling for 6 weeks. Active assisted ROM exercises started at four weeks following surgery. Sub-maximal isometric exercises started at six weeks post-operatively. Active exercises in all directions started at 6 weeks in lying progressing to upright position at 7 weeks. Resistive exercises involving theraband started at 12 weeks.

### Statistical analysis

Descriptive statistics [means, standard deviation (SD)] were calculated for the variables of interest. Paired t-tests were conducted to examine change over time in flexion and external rotation and disability measures (ASES, CMS and ShortWORC) in each group. Analysis of Covariance (ANCOVA) examined the impact of individual factors (age as continuous data, sex as male/female, mechanism of injury as categorical data, having a rotator cuff repair, an endocrine condition (diabetes, thyroid or hypothyroid), and a work-related compensation claim as yes/no on post-op ROM (flexion and external rotation) scores while adjusting for pre-op ROM. Statistical analysis was performed using SAS® version 9.1.3 (SAS® Institute, Cary, NC). Statistical results are reported using 2-tailed p values with significance set at *p <* 0.05.

### Surgical procedures

All surgical procedures were performed by the senior surgeon (RH), with the patient under general anesthesia in the lateral position, and the surgical arm in balanced traction. Patients with impingement (diagnosed clinically using impingement signs, and having Bigliani type II/III acromial morphology on the supraspinatus outlet radiograph) were treated with acromioplasty and coraco-acromial ligament release. Arthroscopic resection of distal clavicle was performed for moderate and severe osteoarthritic changes of the acromioclavicular joint, as diagnosed on Zanca radiograph, and confirmed intra-operatively. Patients with full-thickness tears of the rotator cuff had arthroscopic repair of the tendon(s). Partial tears of the long head of the biceps up to 50 % through the tendon were debrided, and tears more than 50 % were treated with tenodesis or tenotomy. No patient underwent manipulation under anesthesia or formal capsular release procedures.

## Results

Review of data identified 541 patients who had undergone rotator cuff related shoulder surgery between the years 2004–2014. Of 541 patients, 218 patients (54 females, 164 males, mean age: 60, SD:11, age range: 24–85) met the diagnostic criteria for stiffness under anesthesia in at least one direction. There were 26 patients in group 1 (stiffness in both directions), with 192 in group 2 (stiffness in one direction only), of whom 19 had isolated restricted flexion and 173 had isolated restricted external rotation.

Of the total 218 patients, 145 had an arthroscopic rotator cuff repair and 73 patients underwent arthroscopic acromioplasty. Thirty-one (14 %) patients had an active workers’ compensation claim related to their shoulder joint. Sixty-eight (31 %) had an insidious onset, with 150 (69 %) having a history of traumatic or repetitive injury. An endocrine-related comorbidity was reported by 31 patients (14 %).

### Range of motion and disability

**Fig. 1:**
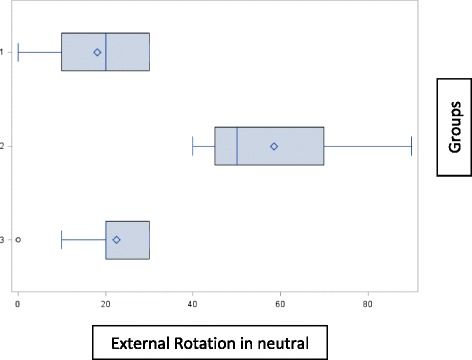
Category boundaries of external rotation for each group

Figure 1 shows the boundaries of ROM in external rotation for each group. Table 1 shows the pre, post and average change in ROM for different directions of stiffness. For the entire group as a whole, there was a statistically significant improvement in ROM in both directions at 6 months post-operatively (*p <* 0.0001). Patients in group 1 who were stiff in flexion and external rotation improved in both directions of flexion and external rotation as well as all disability outcome measures at six months (*p <* 0.0001). Patients with isolated restricted external rotation improved in both directions and all disability outcome measures at 6 months (*p <* 0.0001). Patients with isolated restricted flexion and normal external rotation improved in flexion (*p <* 0.0001) with no further change in external rotation (*p =* 0.43). This group showed a statistically significant (p values varying from 0.002 to 0.0005) improvement in all disability outcome measures at six months as well (Table 2).

**Table 1:** Pre and post-operative range of motion of three groups

Variables	Group1: *N =* 26	Group2a: *N =* 19	Group2b: *N =* 173
Mean (SD)			
Flex			
Range: 0–180°			
Pre	96(5)	95(5)	149(24)
Post	142(22)	134 (37)	155(21)
Post-pre	45(21)	37(32)	26 (31)
Change^a^	*p <* 0.0001	*p <* 0.0001	*p <* 0.0001
ER			
Range: 0–90°			
Pre	19(10)	59(17)	22(8)
Post	44(20)	56(18)	52(16)
Post-pre	25(16)	−3.16 (17)	30(17)
Change^a^	*p <* 0.0001	*p =* 0.43	*p <* 0.0001

*Flex* Flexion

*ER* External Rotation

Change^a^ refers to subgroup analysis using paired t-tests

Group1: Restriction of motion in both flexion and external rotation (Flex ≤100 & ER ≤30)

Group2a: Restricted flexion (Flex ≤100)

Group2b: Restricted external rotation (ER ≤30)

**Table 2:** Pre and post-operative disability scores of three groups

Variables	Group1: *N =* 26	Group2a: *N =* 19	Group2b: *N =* 173
Mean (SD)			
ASES:			
Range: 0–100			
Pre	40(19)	42(24)	51(19)
Post	75(16)	70(24)	77(21)
Post-pre	36(20)	28(27)	27(23)
Change^a^	*p <* 0.0001	*p =* 0.0003	*p <* 0.0001
RCMS:			
Range:0–100			
Pre	36(18)	42(19)	52(20)
Post	76(24)	73(35)	82(24)
Post-pre	40(20)	31(31)	30(23)
Change^a^	*p <* 0.0001	*p =* 0.002	*p <* 0.0001
SHORTWORC			
Range:0–100			
Pre	34(18)	35(19)	42(21)
Post	71(24)	62(30)	71(26)
Post-pre	35(21)	27(28)	29(28)
Change^a^	*p <* 0.0001	*p =* 0.0005	*p <* 0.0001

*ASES* American Shoulder and Elbow Surgeons

*SHORTWORC* SHORT Western Ontario Rotator Cuff

*RCMS* Relative Constant Murley Score

Change^a^ refers to subgroup analysis using paired t-tests

Group1: Restriction of motion in both flexion and external rotation (Flex ≤100 & ER ≤30)

Group2a: Restricted flexion (Flex ≤100)

Group2b: Restricted external rotation (ER ≤30)

Both repair and decompression groups improved in their range of motion of flexion and external rotation at 6 months (*p <* 0.0001) with no statistically significant group differences. Being a female, having a traumatic injury, or having an endocrine illness, did not have a negative impact on recovery of range of motion in any direction. The ANCOVA showed a relationship between age and recovery of external rotation (F_2,216_ = −5.78, *p =* 0.02) with older patients showing less improvement. However, age was not related to recovery of flexion. Patients with an active worker’s compensation claim had similar pre-operative flexion restrictions (120° vs. 123°, *p >* 0.05) but demonstrated a lower level of improved flexion post-operatively compared to those without an active compensation claim (140° vs. 153°, F_2,216_ = −8.76, *p =* 0.003). This difference did not apply to external rotation, which improved from pre-operative range of (24° vs. 26°, *p >* 0.05) to (47° vs. 52°, *p >* 0.05) respectively.

## Discussion

The most important finding of this study is that patients suffering from RC pathology with pre-operative restricted passive ROM improve in the restricted direction at six months following surgery. This is clinically important as patients with the limited ROM can be assured that improvement in passive ROM can be generally expected after surgery without further procedures. The question of whether this improvement is secondary to post-operative reduced pain and ability to better rehabilitate stiffness or improved capsular contracture secondary to change in kinematics of acromioclavicular or glenohumeral joints following decompression or repair of RC remains to be answered.

The sex of the patient, having a full thickness RC tear requiring a repair, presence of diabetes/other endocrine conditions, or having a traumatic injury did not have a negative influence on post-operative ROM in our sample. However, older patients did not tend to recover in their external rotation as well as younger patients. A possible explanation for this may be that age related changes in the collagen of the anterior capsule (non-enzymatic glycosylation and increased cross-linking), [27] lead to an associated decrease in elasticity, and an increase in mechanical stiffness. These changes may become irreversible over time, explaining why this movement is not improved significantly. An inferior recovery of isolated flexion in injured workers with an active compensation claim is interesting and may be related to being apprehensive or resistant to the examiner in this specific direction, potentially due to non-physical reasons, but this needs to be further investigated in future studies.

To our knowledge, only one study has focused upon preoperative stiffness in patients undergoing arthroscopic rotator cuff repair without using manipulation under anesthesia or capsular release [19]. Consistent with our study, Taura [19] reported that mild and moderate stiffness resolved following rotator cuff repair and routine therapy. The author [19] retrospectively categorized 72 patients with full-thickness tears undergoing arthroscopic rotator cuff repair into having a mild (0° to 20°), moderate (20° to 70°), or severe (70°) deficit in total preoperative ROM. At a minimum of two year follow-up, the mean total ROM deficit decreased from 10° to 4° in the mild group, 36° to 12° in the moderate group, and 89° to 31° in the severe group. In this study [19] only three patients who had a RC tear and true adhesive capsulitis required a secondary capsular release.

## Limitations

This study has some limitations that are inherent in retrospective studies and its findings should be interpreted in light of these limitations. As a secondary analysis of prospectively collected data, the research questions were formed retrospectively. The definition of stiffness in this study is somewhat subjective, but we have used it to represent a limitation in functional ROM. This study is generalizable to patients with associated rotator cuff disease and the findings may not be applicable to other shoulder conditions. This study used examination under anesthesia by a single senior surgeon to provide a more accurate estimate of passive ROM by eliminating the impact of pain and muscle spasm, while the postoperative ROM assessment was made by a physiotherapist with the patients awake. The type of examination may introduce some variation. However, in patients with stiffness, there is a high probability that pre-operative passive ROM is more restricted due to pain and spasm. Similarly, passive ROM is expected to be less restricted under anesthesia, should this was feasible post-operatively, which in both cases would overestimate the results of surgery. While the follow up is only six months, this time period was chosen because, it is expected that by this time most patients have made the majority of their recovery in regards of range of motion.

## Conclusion

Patients with RC pathology and concomitant stiffness showed significant improvement in ROM at six months following RC decompression or repair without the need for formal capsular releases or the performance of manipulation under anesthesia. Being a female, having a traumatic event, RC repair, or suffering from an endocrine illness such as diabetes, did not have a negative impact on recovery. However, older patients and those with active Workers Compensation claim showed an inferior recovery in isolated directions.

## Abbreviations

ASES: American shoulder and elbow surgeon’s form
CMS: relative constant murley score
RC: rotator cuff
ROM: range of motion
ShortWORC: short western ontario rotator cuff index
